# Efficacy and safety of S‐flurbiprofen plaster in knee osteoarthritis patients: A 2‐week randomized controlled Phase III clinical trial compared to diclofenac gel

**DOI:** 10.1111/1756-185X.14307

**Published:** 2022-02-23

**Authors:** Kenji Tomatsu, Shoji Yasuda, Ahmad Fuady, Hideo Matsumoto, Sumariyono

**Affiliations:** ^1^ International Business Headquarter Taisho Pharmaceutical Co., Ltd. Tokyo Japan; ^2^ Department of Community Medicine Faculty of Medicine Universitas Indonesia Jakarta Indonesia; ^3^ Public Interest Incorporated Foundation Japan Sports Medicine Foundation Tokyo Japan; ^4^ Department of Internal Medicine Faculty of Medicine Universitas Indonesia – Cipto Mangunkusumo Hospital Jakarta Indonesia

**Keywords:** nonsteroidal anti‐inflammatory drug, osteoarthritis, pain, patch, randomized controlled study, S‐flurbiprofen, visual analog scale

## Abstract

**Aim:**

S‐flurbiprofen plaster (SFPP) is a novel topical nonsteroidal anti‐inflammatory drug (NSAID) patch. This study aimed to assess the efficacy and safety of SFPP in knee osteoarthritis (OA) patients compared to diclofenac gel.

**Methods:**

This study was a multicenter, randomized, active‐controlled, open‐label, non‐inferiority phase III trial. There were 311 enrolled patients treated by SFPP or diclofenac gel for 2 weeks. The primary efficacy outcome was the knee pain when rising from the specially arranged chair assessed by visual analog scale (rVAS). The other efficacy outcomes were clinical symptoms, pain on walking, global assessment by both investigator and patient, and use/non‐use of the rescue drugs during the treatment period. Adverse events (AEs) were evaluated as the safety outcome.

**Results:**

The least‐squares mean (95% CI) of ΔrVAS at the end of the study was 41.52 (39.16‐43.88) mm in the SFPP group and 36.01 (33.69‐38.33) mm in the diclofenac gel group, with a between‐group difference of 5.51 (2.20‐8.82), indicating non‐inferiority. There were statistically significant differences between the groups in rVAS, clinical symptoms, pain on walking, and the global assessment by both investigator and patient. The incidence rate of AEs in the SFPP group was 5.8%, and there was no statistically significant difference from that in the diclofenac gel group (5.2%). Most of the AEs were mild, and no AE led to discontinuation.

**Conclusion:**

Non‐inferiority of SFPP to diclofenac gel was demonstrated in the efficacy for pain on rising from a chair. SFPP was also well‐tolerated in knee OA patients.

## 1 INTRODUCTION

Osteoarthritis (OA) is the most common joint disorder characterized by chronic pain, inflammation and impaired overall functioning.^1^ The incidence of OA is increasing by rising aged population, and it affects more than 500 million people worldwide.^2^

Because the main symptom of OA is chronic pain, it is often treated using nonsteroidal anti‐inflammatory drugs (NSAIDs) in both oral and topical formulations. As oral NSAIDs are recognized to have a risk for gastrointestinal side effects,^3^ topical NSAIDs are often used as its alternatives.^4^ Recently, several OA guidelines and systemic reviews strongly recommend topical NSAIDs for OA treatment.^4^, ^5^, ^6^, ^7^, ^8^, ^9^, ^10^ However, the main concern with topical NSAIDs is lower efficacy than oral NSAIDs as they are considered to have a lower absorption rate.^11^

To solve this concern, S‐flurbiprofen plaster (SFPP), a novel topical NSAID patch was developed. The plaster contains S‐flurbiprofen (SFP), the active form of the widely used racemic flurbiprofen (FP). SFP has a potent inhibitory action on cyclooxygenase (COX) and is anti‐inflammatory and analgesic.^12^, ^13^ SFPP is a tape‐type patch with superior percutaneous absorption formulation, which allows sufficient deep‐tissue penetration of SFP.^14^ Several previous studies showed that SFPP could relieve pain significantly compared to placebo and FP patch,^15^, ^16^ and has an acceptable safety profile in long‐term use.^17^, ^18^, ^19^

Currently, diclofenac gel is recognized as one of the most common topical NSAID agents used for knee OA.^20^ Diclofenac gel can predominantly inhibit COX‐2 enzymes and reduce prostaglandin production. It also has various evidence for OA treatment including head‐to‐head clinical trials with oral NSAIDs.^21^, ^22^, ^23^ Therefore, we conducted a clinical trial to assess the efficacy and safety of SFPP in knee OA patients compared to diclofenac gel.

## 2 METHODS

### 2.1 Study design

This study was a multicenter, randomized, active‐controlled, open‐label, non‐inferiority phase III study to evaluate efficacy and safety of SFPP in knee OA patients (NCT03434197, https://clinicaltrials.gov/ct2/show/NCT03434197). Diclofenac gel, which contains 11.6 mg of diclofenac diethylamine per gram, was selected as an active comparator, as this dose is used widely in clinical situations. Two‐week application period was determined following the European Medicines Agency guideline and previous SFPP and diclofenac gel studies.^15^, ^16^, ^24^, ^25^

### 2.2 Subjects

**FIGURE 1:**
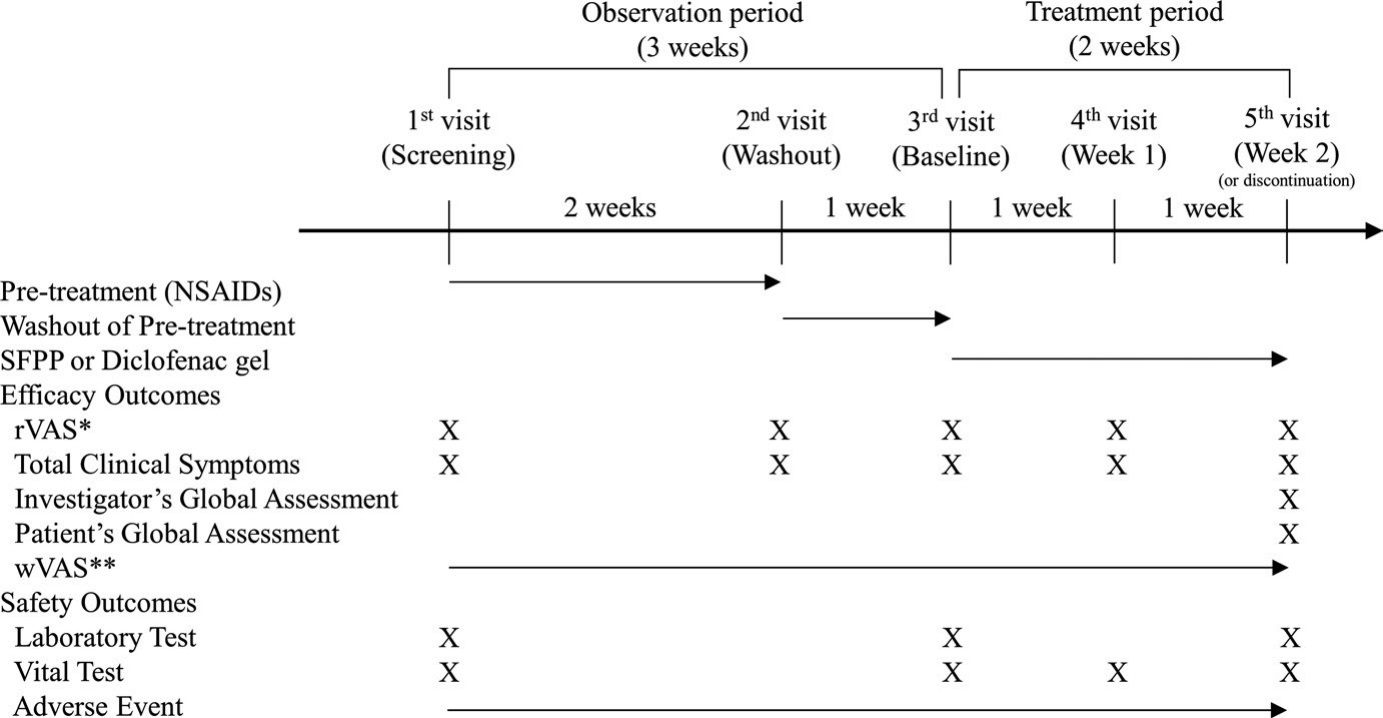
The flow of the study. *Knee pain on rising from the chair assessed by visual analog scale (rVAS). **Knee pain on walking assessed by VAS (wVAS). NSAIDs, nonsteroidal anti‐inflammatory drugs; SFPP, S‐flurbiprofen plaster

Knee OA patients with Grade II or III (according to Kellgren‐Lawrence grading) were screened for this study. Those who had unilateral knee pain, understood visual analog scales (VAS), could walk, and were aged ≥40 years at the time of consent were included in this study. In addition, the patients consumed celecoxib 200 mg/d for 2 weeks from the first visit to the second visit as a pre‐treatment (Figure 1), and they were enrolled if they had knee pain when rising from the chair assessed by VAS (rVAS) with the following criteria: <80 mm before washout of pre‐treatment (second visit); ≥40 mm after washout of pre‐treatment (third visit); and a worsening rVAS of ≥15 mm from second to the third visit due to washout of pre‐treatment.

Exclusion criteria were those who: (a) had past surgery in one of the knees; (b) received treatment on the knees including corticosteroids or intra‐articular injection within 6 days before screening; (c) had complications accompanying the knee pain; (d) had a history of hypersensitivity or allergy to NSAIDs; (e) had a history of dermatitis requiring treatment with topical agents; and (f) were pregnant.

The sample size was calculated based on the Phase II study of SFPP compared to placebo and one report on diclofenac gel.^15^, ^25^ From these 2 studies, the difference in the primary outcome (rVAS) between groups was assumed 2 mm in VAS (0‐100 mm), with the typical standard deviation of 18 mm, and non‐inferiority margin of 5 mm (half of the difference between diclofenac gel and placebo in VAS). The significance level of α (one‐sided) was set at .025. Considering the statistical power (1‐*β*) = .9 and estimated excluding rate from analysis of 10%, the number of enrolled subjects necessary for demonstrating the efficacy of SFPP was 156 per group.

### 2.3 Study protocol and outcomes

The subjects were recruited from 11 medical centers (hospitals and clinics) in Indonesia (Jakarta, Bekasi, Tangerang, Bandung, Surabaya, and Malang). The subjects followed a 3‐week observation period to avoid previous treatment bias (see Figure 1). When the subjects were eligible after the observation period, they were randomly allocated into SFPP or diclofenac gel groups in a ratio of 1:1. Then, both groups received their treatment for 2 weeks. In the SFPP group, SFPP containing 40 mg of SFP in a 10 × 14 cm tape‐type patch was applied to the assessed knee once daily, based on the results of previous SFPP studies.^15^ In the control group, 2 g of diclofenac gel were applied to the assessed knee 4 times daily, following the method of administration in the package insert.

The primary efficacy outcome in this study was rVAS, the knee pain when rising from the chair assessed by VAS. This primary efficacy outcome was selected by referring to the previous SFPP clinical studies in Japan.^15^, ^16^ The rVAS assessment was self‐assessed by each subject, and the assessment was performed following the standardized procedure. All study sites used the same chairs and adjusted the seat height for a sitting knee angle of 110° according to the subject’s height. Each subject was instructed to sit on the chair, rest for 5 minutes, and then rise up without any support. rVAS was assessed at every visit, and the changes from baseline visit to Week 1 and Week 2 were calculated.

In addition to rVAS, this study assessed: (a) changes in total clinical symptoms (tCS); (b) investigator's global assessment; (c) patient's global assessment; (d) knee pain on walking assessed by VAS (wVAS); and (e) rescue drugs during the treatment period as secondary efficacy outcomes. This study also assessed: (f) adverse events (AEs); and (g) laboratory tests and vital signs as safety outcomes.

- Changes in tCS: investigators assessed the following 9 components.
  Pain symptoms: (1) pain at passive motion, (2) pain on ascending and descending stairs, (3) rest pain, and (4) tenderness.
  Inflammatory symptoms: (5) swelling of the soft part of the knee joint, and (6) patellar ballottement.
  Impaired activities of daily living (ADL): (7) sitting down, (8) rising, and (9) walking.
    All symptom components were measured by a 4‐point scale (0: absent, 1: mild, 2: moderate, and 3: severe), and the total score was calculated (0‐27).
- Investigator's global assessment: investigators evaluated the improvement in knee symptoms from the baseline visit by the following 5‐point scale (marked improvement, moderate improvement, mild improvement, no change, and getting worse).
- Patient's global assessment: patients evaluated the improvement in knee symptoms by themselves in the same manner as investigator's global assessment.
- wVAS: patients were asked to record their pain in the assessed knee on walking (VAS score) everyday using a patient diary from the screening visit to the end visit.
- Rescue drugs during the treatment period: patients recorded the use of rescue drugs (paracetamol 1000 mg, once daily when patients cannot bear their knee pain) in the patient diary every day.
- AEs: investigators examined AEs throughout the treatment period and determined their causal relationships to the study drug. AEs which had causal relationships to the study drug were defined as adverse drug reactions (ADRs). In addition, the investigator rated the severity of each AE by a 3‐point scale (mild = not interfering with subject's activity, moderate = discomforting to interfere with subject's activities, severe = making subject's activities extremely difficult).
- Laboratory tests and vital signs: investigators performed laboratory tests (hematology, blood chemistry, and urinalysis) and vital signs (blood pressure and pulse rate) according to the schedule shown in Figure 1. Investigators assessed each parameter for the presence of abnormal variations. If an abnormal variation was found, it was reported as an AE.

### 2.4 Statistical analysis

In this study, subjects’ demographics and clinical characteristics were summarized in frequency tables (number and percentages [%]) and their mean and standard deviation values. The differences in the baseline characteristics between groups were confirmed using χ^2^ tests (for gender, Kellgren‐Lawrence grade, and complications), Wilcoxon rank‐sum tests (for age, and weight), and independent *t* tests (for body mass index). Efficacy outcomes with continuous variables (rVAS, wVAS, and clinical symptoms) were analyzed by analysis of covariance (ANCOVA) with treatment groups as the fixed effect and baseline values as the covariate. The least‐squares means and their 95% confidence intervals (95% CIs) in each group were also calculated. Efficacy outcomes with categorical variables (investigator’s and patient's global assessment) were analyzed by Wilcoxon rank‐sum tests. The proportion of ≥50% pain intensity reduction in rVAS and use/non‐use of the rescue drug were analyzed by χ^2^ test, and the total number of days to consume rescue drugs were analyzed by independent *t* test. For safety outcomes, the number of AEs and ADRs were analyzed by using χ^2^ test and Fisher exact test, respectively. Continuous outcomes in laboratory tests and vital signs were analyzed using independent *t* test. The level of significance was set at 5% (2‐sided). All analyses were performed using IBM SPSS version 27.0.

### 2.5 Ethics approval

This clinical study was reviewed and approved by ethics committees selected by the study institutions regarding its conduct from ethical, scientific, and medical perspectives. Investigators obtained written consent from all subjects before participation in the study.

## 3 RESULTS

In this study, 469 knee OA patients were screened, and 311 patients met inclusion criteria for a random assignment to 2 groups: 156 subjects received SFPP, and 155 subjects received diclofenac gel. Thirteen (4.2%) subjects discontinued their participation in this study, and 9 (2.9%) subjects were removed from efficacy analysis due to the significant protocol deviations. Therefore, 289 (92.9%) subjects who had completed the study were included in the efficacy analysis (per‐protocol set) by following International Council for Harmonization Topic E 9 Statistical Principles for Clinical Trials. There was no statistically significant difference in subjects’ demographic and baseline characteristics between the 2 groups (Table 1).

**TABLE 1:** Subjects’ demographic and baseline characteristics

Variables	SFPP	Diclofenac gel	*P*
	143	146	
Gender, n (%)			
Male	16 (11.2%)	21 (14.4%)	.416^a^
Female	127 (88.8%)	125 (85.6%)	
Age, y, mean (SD)	55.92 (8.36)	54.60 (8.36)	.126^b^
Weight, kg, mean (SD)	68.98 (10.72)	70.72 (12.77)	.226^b^
BMI, mean (SD)	29.19 (4.23)	29.77 (4.65)	.316^c^
Kellgren‐Lawrence grade, n (%)			
II	106 (74.1%)	111 (76.0%)	.709^a^
III	37 (25.9%)	35 (24.0%)	
Complications, n (%)			
Present	63 (44.1%)	66 (45.2%)	.844^a^
Absent	80 (55.9%)	80 (54.8%)	
rVAS at the 3rd visit (baseline), mm, mean (SD)	58.31 (11.56)	58.92 (11.19)	.535^b^

Abbreviations: BMI, body mass index; rVAS, pain on rising from a chair assessed by visual analog scale; SFPP, S‐flurbiprofen plaster

^a^ χ^2^ test.

^b^ Wilcoxon rank‐sum test.

^c^ Independent *t* test.

### 3.1 Efficacy

**FIGURE 2:**
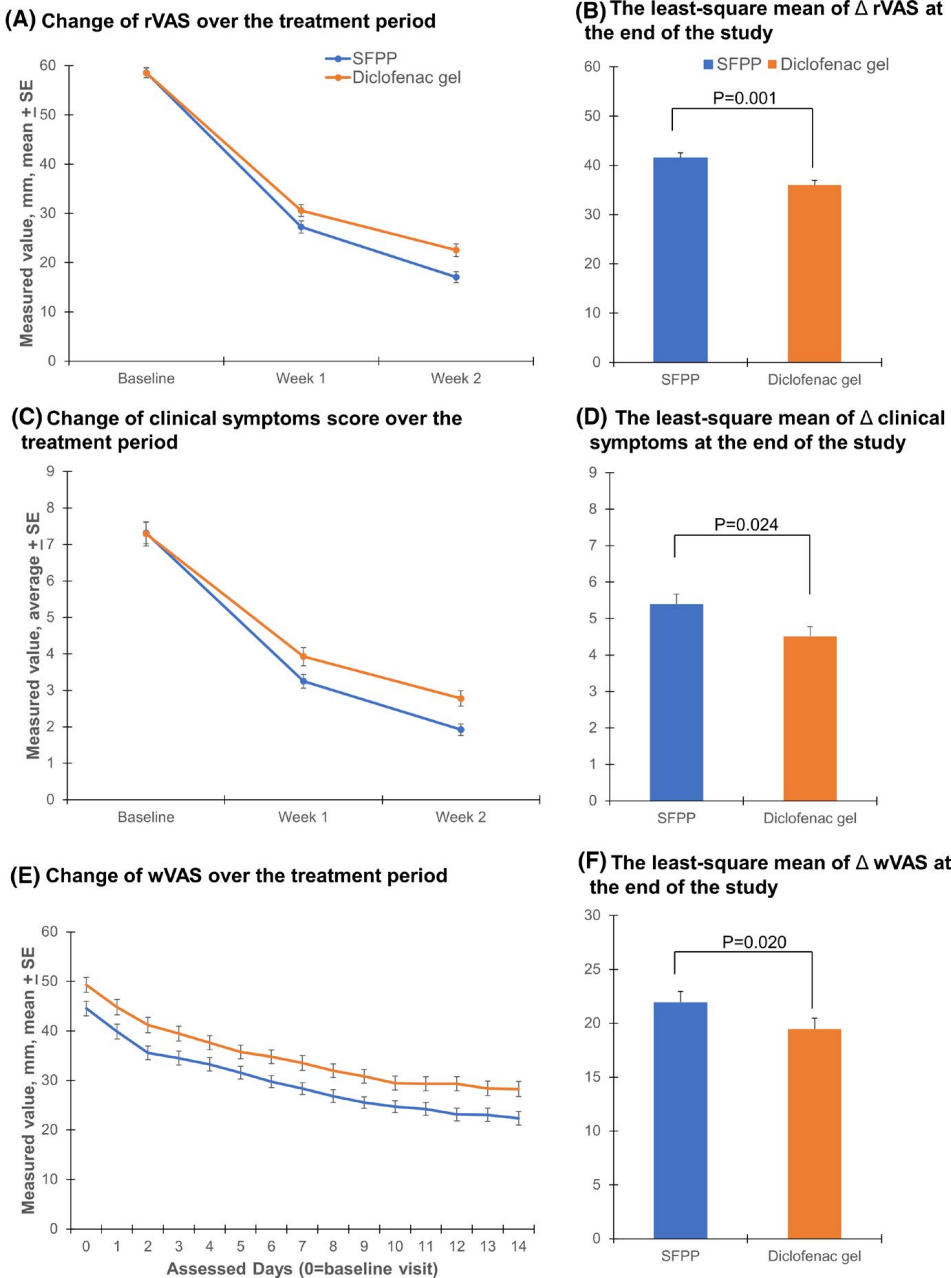
The efficacy of SFPP compared to diclofenac gel in relieving pain indicated by the reduced (A) rVAS, (C) clinical symptoms, and (E) wVAS, and their least‐square mean of (B) ΔrVAS, (D) Δclinical symptoms, and (F) ΔwVAS. SFPP, S‐flurbiprofen plaster; rVAS, pain rising from the chair assessed by visual analog scale; wVAS, pain on walking assessed by visual analog scale

At the end of the study, the least‐squares mean (95% CI) of ΔrVAS was 41.52 (39.16‐43.88) mm in the SFPP group and 36.01 (33.69‐38.33) mm in the diclofenac gel group (*P =* .001). A between‐group difference was 5.51 (2.20‐8.82), indicating non‐inferiority (Figure 2A,B). The proportion of ≥50% pain intensity reduction was 83.8% in SFPP group and 66.7% in the diclofenac gel group (*P =* .001).

At the end of the study, the least‐squares mean (95% CI) of ΔtCS was 5.40 (4.85‐5.94) in the SFPP group and 4.51 (3.97‐5.05) in the diclofenac gel group (*P =* .024) (Figure 2C,D). The least‐squares mean (95% CI) of ΔwVAS was 22.77 (20.31‐25.22) in the SFPP group and 18.68 (16.27‐21.09) in the diclofenac gel group (*P =* .020) (Figure 2E,F).

**FIGURE 3:**
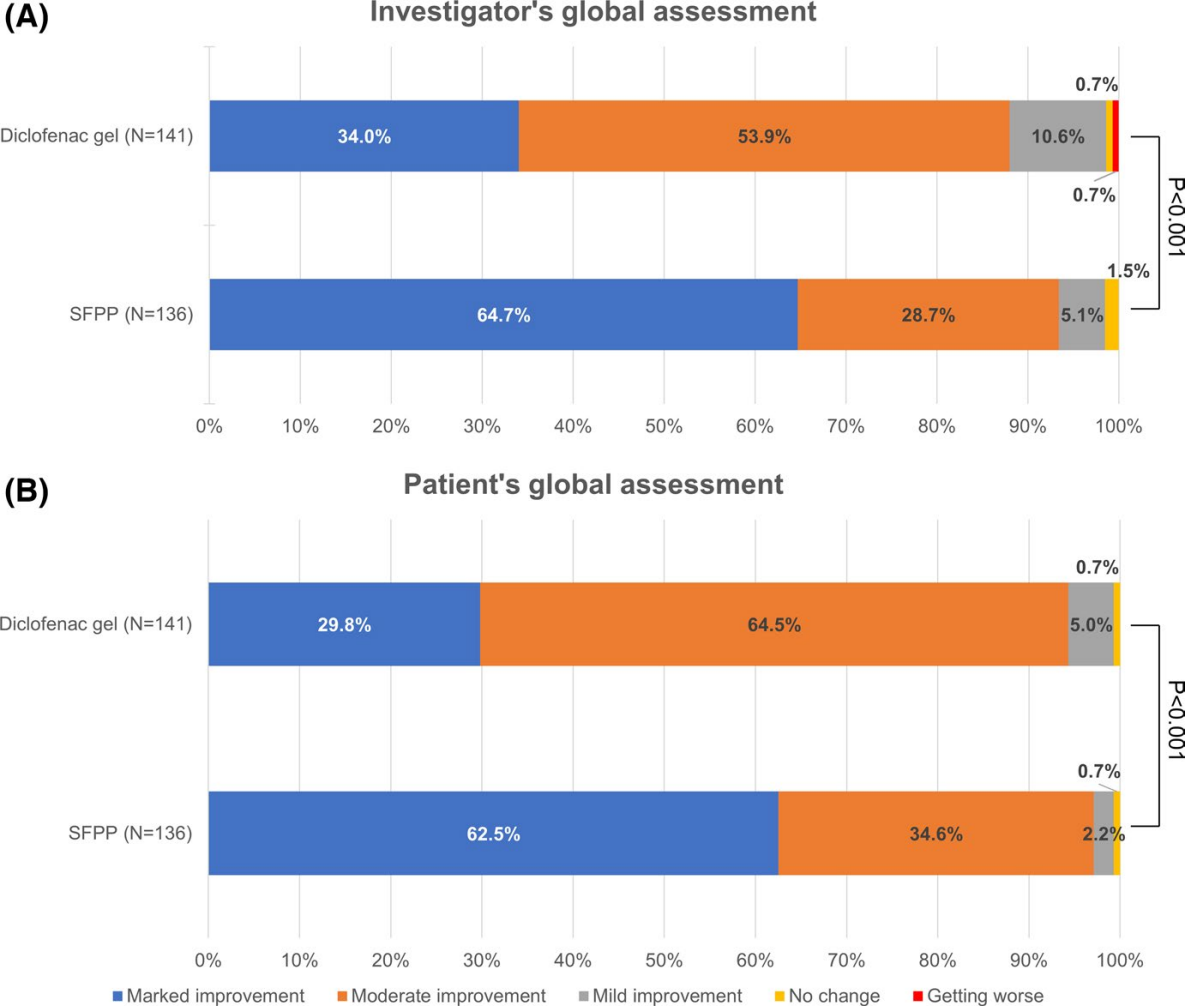
(A) Investigator's and (B) patient's global assessment in S‐flurbiprofen plaster (SFPP) and diclofenac gel groups

The rate of marked improvement in investigators’ and patients’ global assessments were 64.7% and 62.5% in the SFPP group and 34.0% and 29.8% in the diclofenac gel group, respectively. There were statistically significant differences between the groups in both assessments (*P* < .001) (Figure 3).

The number of subjects who used rescue drugs during the treatment period was 54 (38.8%) in the SFPP group and 52 (36.6%) in the diclofenac gel group. The mean total number of days using the rescue drug was 1.09 days in the SFPP group and 0.97 days in the diclofenac gel group. There was no significant difference between the groups.

All primary and secondary efficacy outcomes were analyzed in intention‐to‐treat (311 subjects) as well, and there was no difference in the trend of analysis results compared to the per‐protocol set (289 subjects). With regard to the primary efficacy outcome, the least‐squares mean (95% CI) of ΔrVAS at the end of the study was 40.74 (38.51‐42.97) mm in SFPP group and 35.51 (33.27‐37.75) mm in diclofenac gel group (*P =* .001). A between‐group difference was 5.23 (2.07‐8.39), indicating non‐inferiority.

### 3.2 Safety

For safety analysis, 311 subjects who received study drugs were included. The incidence rates of AEs and ADRs in the SFPP group were 5.8% and 1.9%, and those in the diclofenac gel group were 5.2% and 0.6%. There was no statistically significant difference between the groups. Most of the AEs in both groups were mild, and there was no severe AE. There was also no AE leading to discontinuation (Table 2).

**TABLE 2:** The incidence of AEs and ADRs in both groups

Group	SFPP	Diclofenac gel
Safety population, n	156	155
AE	9 (5.8%)	8 (5.2%)
AEs leading to discontinuation	0	0
ADR	3 (1.9%)	1 (0.6%)
ADR leading to discontinuation	0	0
Name of AEs		
Gastrointestinal disorders^a^	2 (1.3%)	3 (1.9%)
Constipation	1 (0.6%)	0
Diarrhea	1 (0.6%)	0
Nausea	0	2 (1.3%)
Toothache	0	1 (0.6%)
General disorders and administration site conditions^a^	2 (1.3%)	0
Application site dermatitis	1 (0.6%)	0
Application site rash	1 (0.6%)	0
Infections and infestations^a^	4 (2.6%)	2 (1.3%)
Nasopharyngitis	2 (1.3%)	0
Oral candidiasis	0	1 (0.6%)
Otitis media chronic	1 (0.6%)	0
Upper respiratory tract infection	1 (0.6%)	0
Urinary tract infection	0	1 (0.6%)

Abbreviations: ADR, adverse drug reactions; AE, adverse events; SFPP, S‐flurbiprofen plaster

^a^ SOC (System Organ Class as defined by the MedDRA) observed ≥1% in any group.

Two out of the 3 ADRs in the SFPP group were related to the application site. There was only 1 systemic ADR in both groups: one constipation case in the SFPP group and 1 nausea case in the diclofenac gel group.

With regard to laboratory tests and vital signs, statistically significant differences from baseline to Week 2 were detected for red blood cell count, hematocrit and alkaline phosphatase (ALP) in the SFPP group and for hemoglobin, ALP, total bilirubin, lactic dehydrogenase, creatinine, estimated glomerular filtration rate and total protein in the diclofenac gel group. However, the investigators did not consider the change in the laboratory test results in each subject as AE. There was no statistically significant difference from baseline to Weeks 1 and 2 in vital test results in both groups.

## 4 DISCUSSION

### 4.1 Efficacy

From the results of this study, the strong efficacy of SFPP in relieving pain was demonstrated. In addition to demonstrating the non‐inferiority of SFPP compared to diclofenac gel, this study also observed statistically significant differences in ΔrVAS, ΔwVAS, ΔtCS and both investigator’s and patient's global assessments between groups. These findings were consistent with previous trials, which showed that SFPP 40 mg had a remarkable effect on pain relief compared to placebo and flurbiprofen patch.^15^, ^16^ Another recent study also showed the non‐inferiority of SFPP monotherapy to the conventional treatment with a combination of oral NSAIDs (such as celecoxib) and topical NSAIDs patch (such as ketoprofen) in pain assessment (VAS) after a 2‐week evaluation.^26^ Pain is the main symptom of OA, and it greatly impairs quality of life in patients.^27^ Therefore, SFPP, which has a strong effect on pain, is considered to be very suitable for OA treatment.

The difference in the efficacy between SFPP and diclofenac gel in this study can be considered caused by the difference in percutaneous absorption rate. SFPP has a high percutaneous absorption rate (50%‐70%) and can effectively penetrate the deep tissue of the knee joint (synovial tissue and fluid).^14^ The penetration of SFPP is considerably better than those of other topical NSAID patches, including ketoprofen and loxoprofen,^12^ as well as FP patch.^14^ As the comparator in this study, diclofenac gel can also penetrate subdermal tissues, including the synovial tissue.^20^ However, the reports of the concentration of diclofenac within deep tissues after topical administration are variable and not consistent.^20^ The formulation of topical NSAIDs is known to significantly affect their percutaneous absorption.^28^ The absorption rate of diclofenac gel used in this study was reported as 6%,^29^ which is much lower than that in SFPP.

### 4.2 Safety

This study highlighted that SFPP has no apparent concern for safety. The most common AE associated with topical NSAIDs is local skin irritation or application site reactions.^30^ In the SFPP group, only 2 subjects experienced ADR in the application site, and both were mild. These local ADRs were less likely related to S‐flurbiprofen because the previous report revealed no consistent trend of the local ADRs depending on SFPP doses.^15^ Instead, the local ADRs were more likely associated with physical stimulation, such as irritation upon patch removal.^31^ The lower incidence of application site‐related AEs in this study, compared to those in the Japan study (9.5%),^16^ may be related to the differences in subjects’ characteristics. Skin barrier function is greatly affected by aging,^32^ and the subjects in this study were younger than those in the Japan study (67 years on average). In addition, the environmental factors in Indonesia, such as the higher temperature and humidity, may also contribute to the lower incidence of application site‐related AEs.^33^

Besides the low incidence of local ADRs, SFPP also demonstrated the low incidence of ADRs related to gastrointestinal disorders (1 constipation case only). Although systemic exposure in SFPP is higher than those in conventional topical NSAIDs due to the high percutaneous absorption, the transdermal patches, including SFPP, have no direct effect on gastric mucosal epithelial cells.^34^, ^35^, ^36^ As a result, the risk of gastrointestinal disorder in SFPP was similar to that in diclofenac gel.

### 4.3 Limitation

This study has some limitations. First, this study was designed as an open‐label study because SFPP and diclofenac gel dosage forms are different. Investigators could recognize the type of study drug (patch or gel) during the efficacy and safety assessment. Thus, expectation bias on efficacy outcomes remains. To reduce the bias, investigators were blinded until the study drugs were allocated, and the subjects, who assessed the primary efficacy endpoint (rVAS) by themselves, were not informed which study drug (patch or gel) is a test drug.

Second, this study did not use Western Ontario McMaster Universities Osteoarthritis Index (WOMAC) knee pain score as the primary outcome, which is widely used in OA clinical studies. The results, therefore, may not be comparable with other trials using WOMAC outcome. However, rVAS was set as a primary efficacy endpoint because “knee pain on rising from the chair” is one of the major symptoms of knee OA, and rVAS is considered to be reproducible by using the same chair and a standardized procedure. Therefore, we determined that rVAS is valid, and the efficacy endpoints in this study could adequately assess efficacy of SFPP for knee OA pain instead of WOMAC.

And third, we evaluated the efficacy and the safety of SFPP in a 2‐week study but have not investigated those in long‐term use. Although the efficacy results in this study were consistent with the previous reports, the safety results from this study suggested that patients in South‐East Asia may have a lower risk of developing skin AEs at the application sites than Japanese patients. According to the long‐term clinical study in Japan, the 52‐week application of SFPP was still well‐tolerated. However, the incidence of the skin‐related AEs at the application site was higher in the long‐term study than in the 2‐week studies.^16^, ^19^ It is necessary to evaluate the tolerability of SFPP for South‐East Asian patients in long‐term use as well.

## 5 CONCLUSION

Non‐inferiority of SFPP to diclofenac gel was demonstrated in the efficacy for pain on rising from a chair. There were statistically significant differences between the groups in most efficacy outcomes, indicating the robust efficacy of SFPP for knee OA patients. SFPP was also well‐tolerated in knee OA patients.

In addition, these efficacy results were obtained by once‐daily application of SFPP, although topical NSAID gels including diclofenac gel need to be applied multiple times per day. This new topical NSAID patch will improve both medication adherence and the quality of life for OA patients.

## CONFLICT OF INTEREST

K. Tomatsu and S. Yasuda are employees of Taisho Pharmaceutical Co., Ltd. A. Fuady received a statistical analysis fee from Taisho Pharmaceutical Co., Ltd. H. Matsumoto received a consultant fee from Taisho Pharmaceutical Co., Ltd. Sumariyono received an investigator fee from Taisho Pharmaceutical Co., Ltd.

## AUTHOR CONTRIBUTIONS

K. Tomatsu contributed to study design, study management, interpretation of data, and writing the manuscript. S. Yasuda contributed to study management and interpretation of data. A. Fuady contributed to statistical analysis and writing the manuscript. H. Matsumoto contributed to study design and interpretation of data. Sumariyono contributed to study site management and interpretation of data. All authors reviewed and approved the final version of the manuscript.
